# Maternal exposure to fine particulate matter during pregnancy induces progressive senescence of hematopoietic stem cells under preferential impairment of the bone marrow microenvironment and aids development of myeloproliferative disease

**DOI:** 10.1038/s41375-019-0665-8

**Published:** 2019-11-27

**Authors:** Govinda Bhattarai, Jae Bong Lee, Min-Hye Kim, Suhan Ham, Han-Sol So, Sangmin Oh, Hyun-Jaung Sim, Jeong-Chae Lee, Mijung Song, Sung-Ho Kook

**Affiliations:** 1 Department of Bioactive Material Sciences, Research Center of Bioactive Materials, Chonbuk National University, Jeonju, 54896 South Korea; 2 Institute of Oral Bioscience and School of Dentistry, Chonbuk National University, Jeonju, 54896 South Korea; 3 Thermal Hydraulics and Severe Accident Research Division, Korea Atomic Energy Research Institute, Deajeon, 34057 South Korea; 4 Department of Earth and Environmental Sciences, Chonbuk National University, Jeonju, 54896 South Korea

**Keywords:** Haematopoietic stem cells, Biological sciences, Stem cells

## To the Editor:

During the last few decades, industrial development and expansion have led to significantly increased levels of fine particulate matter (PM) in the air, including particles with aerodynamic diameters smaller than 2.5 μm (referred to as PM_2.5_) [1]. PM_2.5_ can have negative effects on air quality and threatens human health. Furthermore, it can have effects on tissue-specific stem progenitor cells [2, 3]. However, little is known about the mechanisms underlying the effects of PM_2.5_ on stem progenitor cells, particularly hematopoietic stem progenitor cells (HSPCs). There is growing evidence that maternal exposure to PM_2.5_ during pregnancy can harm both the embryo and progeny [4–6]. The embryo and fetus are more susceptible to external stress than the adult. Although there is growing evidence regarding the detrimental risks to the embryo and offspring that have been maternally exposed to PM_2.5_ during pregnancy [4–6], little is known about the effects of maternal PM_2.5_ exposure to stem cells, which begin to emerge, activate, and mature during embryo development.

In this study, we developed an atmospheric simulation chamber (ASC) (Supplementary Fig. 1a), and demonstrated that maternal exposure to PM_2.5_ (2 h on five consecutive days, ~50 μg/m^3^ PM_2.5_ mass concentration, Supplementary Tables 1 and 2, Supplementary Fig. 1b, c) during pregnancy can affect the lungs of the fetus. The PM_2.5_ that reached the fetal lungs incurred oxidative stress and inflammation, in agreement with previous reports (Supplementary Fig. 1d) [7, 8]. The initially detrimental phenomena in the fetal lungs that were triggered by maternal exposure of PM_2.5_ continued after birth, leading to impairment in the bronchiole structure of the offspring born to the PM_2.5_-exposed dam (Supplementary Fig. 1e). A greater number of cells, including CD4^+^ and CD8^+^ T cells, infiltrated the bronchioles of PM_2.5_-exposed offspring, which also exhibited mild fibrosis (Supplementary Fig. 1f, g). The NLRP3 inflammasome, one of the most fully characterized inflammasomes, has been implicated in the pathogenesis of inflammatory and fibrosis diseases. A previous study confirmed that PM_2.5_ could activate the NLRP3 inflammasome [9]. Indeed, we verified the upregulation of NLRP3 in the lung tissues of PM_2.5_-exposed offspring. The results further confirmed that PM_2.5_ promoted the activation of cleaved caspase-1 and IL-1β, which are representative markers of inflammasome activation (Supplementary Fig. 1h, i). Besides the lung, oxidative stress and inflammation were also observed in other tissues (Supplementary Fig. 2a) such as the liver, brain, kidney, spleen, and thymus of the PM_2.5_-exposed offspring. However, there was no severe inflammation and oxidative stress in the fetus liver (FL) and brain and also no alteration in bone development in the fetus (Supplementary Fig. 2a–c). These results indicate that maternal exposure to fine PM_2.5_ during pregnancy can also detrimentally affect other tissues of the offspring by inducing systemic inflammation, but not the fetus.

**Fig. 1:**
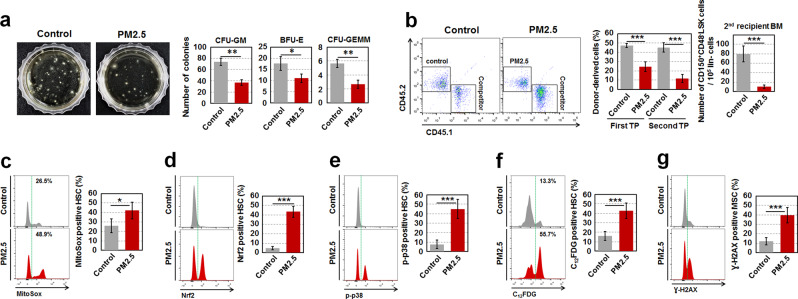
Maternal exposure to fine PM_2.5_ during pregnancy leads to the progressive senescence of HSCs in offspring of 6 months. **a** For the colony assay, BM cells (2 × 10^4^ per dish) in PM_2.5_-exposed offspring of 6 months were incubated in a methylcellulose-based medium for 12 days and the colonies formed were counted. Representative data are shown for three independent experiments. **b** For long-term competitive repopulating activity, equal numbers (5 × 10^5^) of BM cells from control or PM_2.5_-exposed offspring were co-transplanted with those from competitor mice (CD45.1) into lethally irradiated recipient mice (CD45.1/2, 1000 rads, *n* = 7) that were also transplanted with BM cells (1 × 10^6^) of CD45.1/2 mice after the first transplantation. PB was collected from the recipient mice at 4 months post-transplantation and the ratio of CD45.1/CD45.2 was assessed by flow cytometry. Donor-derived HSCs engrafted in the BM of the secondary recipient mice were measured by flow cytometry after the procedure of lineage cell depletion (*n* = 4). **c** Levels of mitochondrial superoxide anions in the BM HSCs of offspring of 6 months were measured with MitoSOX™ Red reagent using flow cytometry (*n* = 11). Levels of Nrf2 (**d**) and p38 phosphorylation (**e**) were analyzed in the BM HSCs of the offspring after the fixation and permeabilzation procedure (*n* = 7). **f** SA-β-gal activity in BM HSCs of the offspring were measured using incubating the cells with C_12_FDG, a β-galactosidase substrate (*n* = 11). **g** Levels of γ-H2AX were analyzed in the BM HSCs of the offspring after the fixation and permeabilization procedure (*n* = 5). All data are presented as the means ± SD. *p < 0.05, **p < 0.01, and ***p < 0.001 vs. control, as determined by Student’s *t* tests

However, maternal exposure to PM_2.5_ did not directly affect FL hematopoietic stem cell (HSCs), which serves as a main site for the expansion and differentiation of HSCs during fetal life, until hematopoiesis shifts to the bone marrow (BM) around birth (Supplementary Fig. 3a, b). No detrimental effects of maternal PM_2.5_ in FL HSPCs carried over to the BM HSPCs in offspring of 2 months, which were born to PM_2.5_-exposed pregnant dam (Supplementary Fig. 3c–f). Of note, PM_2.5_-exposed offspring of 6 months exhibited senescent phenotypes in BM HSCs, as evidenced by lower clonogenic formation, donor cell-derived reconstitution, and self-renewal, as well as higher levels of mitochondrial ROS, Nrf2 expression, p38 phosphorylation, SA-β-gal activity, and biased-myeloid differentiation (Fig. 1a–f and Supplementary Fig. 4a–h) [10, 11]. mRNA levels of cyclin-dependent kinase inhibitors such as *p16*, *p21*, *p19*, and *p15* also increased in BM HSCs of PM_2.5_-exposed offspring compared with those of control offspring (Supplementary Fig. 4i). PM_2.5_-exposed offspring-derived BM HSCs exhibited higher levels of DNA double-strand breaks compared with their control offspring-derived counterparts, as demonstrated by flow cytometry using a γ-H2AX antibody (Fig. 1g). However, there were no significant differences in the numbers of circulating WBCs, RBCs, and platelets between control and PM_2.5_-exposed offspring (Supplementary Fig. 4j). These results led us to hypothesize that, although HSCs are not directly affected by maternal exposure to PM_2.5_ during pregnancy, they may be progressively altered to be senescent under the abnormal BM microenvironment formed via non-cell autonomous processes. As expected, the exposure to fine PM_2.5_ during pregnancy impaired the BM microenvironment by inducing ROS-mediated senescence of bone cells and MSCs in offspring of 2 months (Supplementary Fig. 5). A previous report indicated that age-associated bone cell senescence enhances osteoclastogenesis [12]. Our findings were consistent with these results, as PM_2.5_-exposed offspring of 2 months exhibited a high level of osteoclast activity, supported by relatively increased RANKL to OPG levels and high levels of inflammatory cytokines (Supplementary Fig. 6a–g). An age-related BM microenvironment with preferential osteogenesis and senescent osteoblastic cells in the PM_2.5_-exposed offspring generated high levels of proteolytic enzymes such as MMP-2, MMP-9, and CTK, which are involved in the regulation of HPC retention in the BM (Supplementary Fig. 6h). The preferential impairment of the BM microenvironment in PM_2.5_-exposed offspring led to HSC senescence in non-cell autonomous mechanisms, as evidenced by the induction in senescence and functional defects of donor cell-derived HSCs that were transplanted into conditioned PM_2.5_-exposed offspring recipients (Supplementary Fig. 7). Taken together, these findings demonstrate that maternal exposure to PM_2.5_ during pregnancy preferentially impairs the BM microenvironment, causing it to be aged, after which BM HSCs progressively undergo senescence via non-cell autonomous mechanisms.

The modulating effects of maternal PM_2.5_ exposure on BM microenvironment-mediated HSCs were different from those observed when adolescent mice (4 weeks of age) were exposed to PM_2.5_, as the BM microenvironment and BM HSCs were hardly affected in adolescent PM_2.5_-exposed mice (Supplementary Fig. 8). These results illustrate that PM_2.5_ has different impacts on mice depending on the time at which they are exposed and displays more detrimental effects during the embryo stage than in the adult.

**Fig. 2:**
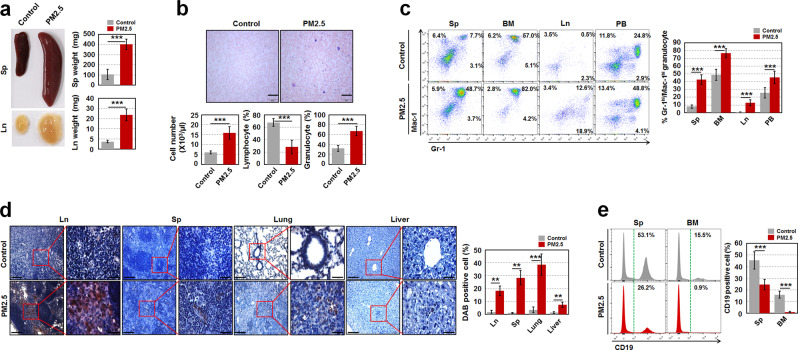
Maternal PM_2.5_-exposed offspring have the potential to develop a myeloproliferative disease. **a** Photographs of the Sp and Ln from control and maternal PM_2.5_-exposed offspring at 1 year of age and weights of those were measured (*n* = 4). **b** May–Grünwald–Giemsa stained blood smear (upper panels) and leukocyte number and the proportion of lymphocytes and granulocytes (lower panels) from the old offspring (*n* = 5). Scale bars are 200 µm. **c** Percentage of Gr-1^+^/Mac-1^+^ granulocytes in Sp, BM, Ln, and PB of the old offspring (*n* = 5). **d** Myeloperoxidase immunohistochemistry was conducted in hematopoietic (Ln and Sp) and nonhemtopoietic organs (lung and liver) of the old offspring to measure infiltrated blast cells to the organs. A representative result is shown (*n* = 5). Scale bars are 100 (left) and 20 (right) µm. DAB-positive cell intensity was measured by ImageJ-win64. **e** Percentage of B cells (CD19^+^ cells) in Sp and BM of the old offspring (*n* = 5). All data are presented as the means ± SD. **p < 0.01 and ***p < 0.001 vs. control, as determined by Student’s *t* tests

Similar to a previous study that showed an aged microenvironment can contribute to aging-related myeloproliferative disease [13], our current findings demonstrated that a total of 9 out of 25 (~36%) PM_2.5_-exposed 1-year-old offspring had the potential to develop a myeloproliferative disease along with increased SA-β-gal activity in MSCs and HSCs during aging (Supplementary Fig. 9), as evidenced by the following: massive enlargement of the spleen (Sp) and lymph node (Ln); increased BM and Sp cellularity; increased number of circulating leukocytes; increased percentage of Gr-1^+^/Mac-1^+^ granulocytes in the Sp, BM, Ln, and PB; higher percentage of immature myeloid cells, such as c-Kit^+^Gr-1^+^ and c-Kit^−^Gr-1^+^ cells; lower percentage of mature myeloid cells, such as c-Kit^+^Gr-1^+^Ly6G^+^ and c-Kit^−^Gr-1^+^ Ly6G^+^ cells; and higher infiltration of myeloperoxidase-stained massive blast cells in the Ln, Sp, and nonhematopoietic organs, such as the lungs and liver in PM_2.5_-exposed old offspring (Fig. 2 and Supplementary Fig. 10).

It has been extensively demonstrated that the inductive generation of oxidative stress by PM_2.5_ exposure is a major determinant in triggering tissue impairment. In this study, we detected increased oxidative stress in the lungs of E16.5, which was immediately observed after maternal PM_2.5_ exposure for five consecutive days. The administration of drinking water that contained NAC to pregnant mice during PM_2.5_ exposure limited the generation of oxidative stress and inflammasomes in the lungs and ameliorated the PM_2.5_-induced impairment of the BM microenvironment, followed by the prevention of BM HSC senescence and normal clonogenic formation (Supplementary Fig. 11). Despite the preventive effects of NAC treatment in PM_2.5_-exposed pregnant mice, the offspring born to those with NAC underwent growth retardation and had a low body weight (data not shown). As a previous report indicated that a slight change in ROS levels is closely involved in various cell signaling processes and can directly affect embryonic development [14], the application of NAC to prevent ROS-mediated detrimental effects in PM_2.5_-exposed mice should be considered during the embryo stage to avert any unexpected additional damage.

The increased detrimental effects of PM_2.5_ exposure to the embryo rather than adolescent mice via the modulation of the BM microenvironment and HSCs, as demonstrated in our findings (Fig. 1 and Supplementary Figs. 3–8) may have been due to an incomplete defense system in the embryo that is unable to prevent PM_2.5_-induced oxidative stress.

In summary, maternal exposure to fine PM_2.5_ during pregnancy destructively affects the fetal lungs by inducing oxidative stress and these harmful effects last to adulthood, followed by the induction of inflammasomes and impaired bronchioles. In addition, maternal exposure of PM_2.5_ causes the progressive senescence of HSCs via the ROS-p38 MAPK and Nrf2 pathway under the exposure-induced preferential impairment of the BM microenvironment with age-related phenotypes. Our results demonstrate that the embryo is more vulnerable to PM_2.5_ exposure than the adolescent, particularly in relation to the BM microenvironment-associated modulation of HSCs. However, only oxidative stress inhibitor experiments have been performed in the present study and show the evidence that the effects of PM_2.5_ were mediated by activation of oxidative stress signaling. On the other hand, further research is needed to investigate the exact role and mechanism of other signaling molecules in the effects of PM_2.5_ by using gene knockout or inhibitor experiments.

## Supplementary information

Supplementary Information
