# Supplementary material for: Maternal exposure to fine particulate matter during pregnancy induces progressive senescence of hematopoietic stem cells under preferential impairment of the bone marrow microenvironment and aids development of myeloproliferative disease
Source: Leukemia. 2019 Nov 27;34(5):1481–4. doi: 10.1038/s41375-019-0665-8 (PMC8076008; doi:10.1038/s41375-019-0665-8)
Supplement: Supplementary file 1 — Supplementary Information [file 41375_2019_665_MOESM1_ESM.doc]

Maternal exposure to fine particulate matter during pregnancy induces progressive

senescence of hematopoietic stem cells under preferential impairment of the bone marrow microenvironment and aids development of myeloproliferative disease

Govinda Bhattarai^#^, Jae Bong Lee^#^, Min-Hye Kim^#^, Suhan Ham, Han-Sol So, Sangmin Oh, Hyun-Jaung Sim, Jeong-Chae Lee, Mijung Song,^*^ and Sung-Ho Kook^*^

**Supplementary Information**

**Materials and Methods**

**Chemical compositions**

The organic components and inorganic salts used in this study are listed in Supplementary Table 1. These organic and inorganic species were selected as model compositions because of their abundance in the troposphere [1–5]. These 10 compounds were mixed with the organic to inorganic dry mass ratio of 1:1 as shown in Table 1 to mimic the chemical complexity of the atmospheric aerosols. All of the compounds were purchased from Sigma-Aldrich with purities ≥ 98%, and were used without further purification. The mixture of 10 components was dissolved in purified water (resistivity ≥ 18.0 MΩ cm).

**Atmospheric simulation chamber system**

The Animal Welfare Committee of Chonbuk National University approved all experimental procedures. The ASC system to expose PM_2.5_ to mice was previously developed and a comprehensive review on ASC can be found in a previous report [6]. The developed ASC system in this study was based on a previously presented method [7]. As shown in Supplementary Fig. 1a, the ACS system is composed of an aerosol generation system, a whole-body chamber, and instrumentation to monitor air quality conditions in the chamber. For PM generation, the chemical solution that contained the dissolved 10 components (in purified water) was atomized using a nebulizer (TQ-50-C0.5; Meinhard, USA) with controlled timing to optimize aerosol concentration. The generated aerosol particles passed through a dilution chamber and were diluted by the introduction downstream of purified air. Then the diluted aerosol particles were passed through a diffusion dryer to strip any remaining moisture. The dried PM was delivered to the mice in a whole-body exposure chamber. Distilled water without the mixture of 10 compounds was used to expose to control mice via ASC system. During the experiment, the PM in the chamber was monitored in real-time using an OPC (OPC-N2; Alphasense, UK) for size distribution and number concentrations. In addition, the mass concentration of PM was evaluated by collecting the aerosols on a filter (47 mm PTFE filter; PALL, USA). Temperature, relative humidity, and oxygen level in the chamber were maintained at 19.3 ± 1.9 °C, 47.0 ± 11.6%, and 21.4 ± 1.4%, respectively.­­­

**PM_2.5_ concentrations**

In the absence of mice, the mean mass concentration of PM_2.5_ in the exposure chamber during 2 h was assessed to be 52.9 ± 11.2 μg/m^3^ by a filter measurement. Details of PM mass concentrations for mice exposure tests are presented in Supplementary Table 2. The PM mass concentration measurement in the absence of mice was performed in triplicate for every batch of mice tested. In addition, the mean concentration of PM_2.5_ in the exposure chamber was estimated from the number concentration measured with OPC, and was observed to be 55.2 ± 10.2 μg/m^3^. Supplementary Fig. 1b shows the time evolution of PM concentration in the exposure chamber in the absence of mice, as measured with OPC. Supplementary Fig. 1c presents a histogram of normalized PM number concentration versus the size range of optical PM diameter for the distribution and demonstrating that the PM in the exposure chamber was PM_2.5_ in the size range.

**Analysis of inorganic salts in PM_2.5_**

Particles were collected at ~10 % relative humidity on a Teflon filter for 2 h in the absence of mice, and the filter was immediately extracted with 5 mL ultrapure water (18.2 mΩ cm at 25 °C). After extraction, the concentration of any inorganic salts on the filter was determined by ion chromatography (ICS-90; Dionex, USA). Using three different filters, the mean mass ion composition was as follows: NO_3_^-^ = 11.7 μg/m^3^, SO_4_^2-^ = 11.9 μg/m^3^, and NH_4_^+^ = 0.96 μg/m^3^. We assumed that the sulfate and nitrate in the extract were fully neutralized by ammonium (in the form of ammonium sulfate and ammonium nitrate respectively). Thus, the mass of ammonium sulfate in the extract was calculated by the sulfate mass × 1.38 and the ammonium nitrate mass was estimated by the nitrate mass × 1.29, resulting in ~16.4 μg/m^3^ for ammonium sulfate and ~15.0 μg/m^3^ for ammonium nitrate. Inorganic salts accounted for ~50% of the PM_2.5_ mass studied in this work.

**Flow cytometry**

The numbers of BM and PB cells collected from mice that had been sacrificed by CO_2_ asphyxiation were measured by multi-color flow cytometry (BD Aria or BD Calibur; BD Biosciences, Franklin Lakes, NJ, USA) installed in the Center for University-Wide Research Facilities (CURF) at Chonbuk National University. The populations of hematopoietic cells from the BM and PB samples were phenotypically gated and further analyzed using the FlowJo software program (FLOWJO; Ashland, OR, USA). BM Lin^-^Sca-1^+^c-Kit^+^ (LSK) cells and CD150^+^CD48^-^LSK HSCs were phenotypically identified using the following antibodies (purchased from BD Biosciences unless specified otherwise): lineage markers phycoerythrin (PE)-Cy7-conjugated anti-CD3, anti-CD4, anti-CD8, anti-CD45R, anti-CD11b, anti-Gr-1, and anti-TER-119; PE-conjugated or fluorescein isothiocynate (FITC)-conjugated anti-Sca-1; allophycocyanin (APC)-conjugated anti-c-Kit; PerCP/Cy5.5-conjuated anti-CD150 (eBioscience); APC–Cy7-conjugated anti-CD48. BM HPCs such as GMP, CMP, MEP, and CLP cells were further characterized using PE-conjugated anti-FcR, PerCP/Cy5.5-conjuated anti-CD34 (BioLegend) on the basis of Lin^-^Sca-1^-^c-Kit^+^ markers, and PE-conjugated anti-IL-7R. FL (E16.5) HSCs were defined using PE-Cy7-conjugated lineage markers that excluded APC-conjugated Mac-1, FITC- or PE-conjugated Sca-1, PerCP/Cy5.5-conjuated anti-CD150 (eBioscience), and APC–Cy7-conjugated anti-CD48 antibodies. MSCs in the BM were phenotypically characterized using same PE-Cy7-conjugated lineages as used for identification of hematopoietic cells, APC-Cy7-conjugated Sca-1, PE- or FITC-conjugated CD26, and APC-conjugated CD105 antibodies. The proportion of circulating monocytes (CD11b^+^) and B-cells (B220^+^) in the PB were measured using FITC- and PE-conjugated antibodies, respectively. BM monocyte cells were also defined using a FITC-conjugated antibody. Gr-1+/Mac-1+ granulocytes in Sp, BM, Ln, and PB were measured using PE-Cy7/FITC-conjugated antibodies. Immature and mature myeloid cells were measured in the PB, BM, and Sp using ACP-conjugated c-Kit, PE-Cy7-conjugated Gr-1, and PE-conjugated Ly6G antibodies. Mitochondrial superoxide anion level and SA-β-gal activity were measured with MitoSox Red (Invitrogen) and C_12_FDG (Molecular Probes), respectively. The protein levels of γ-H2AX (Cell Signaling) and Nrf2 (Cell Signaling) were assessed with the PE- and Alexa Flour 488-conjugated antibody after fixation and permeabilization, respectively. MAPK activation was measured with Alexa Flour 488-conjuated anti-p38 MAPK (BD Biosciences), PE-conjugated anti-JNK (BD Biosciences) and Alexa Flour 488-conjugated anti-ERK (BD Biosciences). To evaluate intracellular ROS levels, BM cells obtained from the control or PM_2.5_-exposed offspring were seeded on 60 mm cultured dishes. After 48 h, cells were incubated with 10 µM DCF-DA (2’,7’-dichlorodihydrofluorescein-diacetate) for 30 min. The green fluorescence of DCF was recorded at 515 nm (FL-1H) using the FACS Calibur® system (Becton-Dickinson, San Jose, CA, USA) and 10,000 events were counted per sample.

**Transplantation**

To evaluate donor cell-derived reconstituting activity, recipient mice (10 Gy) were lethally irradiated 8–24 h prior to transplantation. BM cells (5 × 10^5^) from control or PM_2.5_-exposed offspring (16 weeks old, CD45.2) were co-transplanted with BM cells (5 × 10^5^) from competitor mice (CD45.1) into the conditioned recipient mice (CD45.2) by tail vein injection. To measure the stem cell self-renewal potential, BM cells (1 × 10^6^) from the first transplanted recipient mice were then transplanted into the conditioned recipient mice. Donor-mediated reconstituting activity in the PB of recipient mice was assessed at 16 weeks post-transplantation. To examine the fate of transplanted cells (CD45.1/2) in host mice, normal bone cells (2 × 10^6^, CD45.1/2) were transplanted into sublethally irradiated control or PM_2.5_-exposed offspring (2 months old, 3Gy, CD45.2). PB cells were collected from transplanted host mice at 4 and 8 weeks after post-transplantation, and the ratio of CD45.1/CD45.2 was measured using flow cytometry. At 16 weeks post-transplant, the number of HSCs, mitochondrial ROS level, p38 phosphorylation and SA-β-gal activity of HSCs derived from donor cells (CD45.1/2) were analyzed in the BM of the transplanted host mice (CD45.2). To determine the ability of the donor cells to support the survival of the recipient, BM cells (1 × 10^6^) from control or PM_2.5_-exposed offspring at 6 months of age were non-competitively transplanted into lethally irradiated recipient animals.

**Colony forming cell assay**

FL (E16.5d) and BM cells (2 × 10^4^ per dish) were seeded into 35 mm dishes with MethoCult^Ⓡ^ GF M3434 (Stem Cell Technologies) for the colony-forming unit (CFU) assay. After incubation for 12 d, the numbers of CFU-GM, BFU-E, and CFU-GEMM colonies were counted using standard criteria. To analyze MSC-derived CFU, nucleated BM cells (1 × 10^6^ cells) from the femur and tibia of mice were plated into 6-well plates with αMEM (Welgene Inc.; Daegu, Republic of Korea) consisting of 2 mM glutamine, antibiotics (100 IU/mL penicillin G and 100 μg/mL streptomycin), and 20% fetal bovine serum (FBS). On the second day of incubation at 37 °C in a humidified atmosphere of 5% CO_2_, non-adherent cells were removed, while adherent cells were cultured for a further 12 d in the same medium. At the end of the incubation period, the cells were fixed in 10% formalin for 10 min and then stained with 0.5% crystal violet dissolved in 100% methanol. After washing and air-drying, MSC-derived colonies containing more than 50 cells were counted using an optical microscope.

**Real-time reverse transcription-polymerase chain reaction (RT-PCR)**

Total RNA was extracted with Trizol reagent (Invitrogen Corp., Carlsband, CA, USA) according to the manufacturer’s instructions. RNA samples (1 µg per reaction) from a total of individual group were used for preparation of cDNA for RT-PCR using AmpiGene^TM^ cDNA Synthesis Kit (Enzo Life Sciences, Farmingdale, NY, USA). Power SYBER^Ⓡ^ Green PCR Master Mix (Life Technologie, CA, USA) was used to detect the accumulation of PCR products during cycling with the ABI StepOnePLUS sequence detection system (Applied Biosystems, Foster City, CA, USA) with following thermo-cycle programming: predenaturation at 95 °C for 2 min followed by 40 cycles of denaturation at 95 °C for 5 sec, annealing at 60 °C for 30 sec, and extension at 65 °C for 30 sec. Primer sequences specific to *P15*, *P16*, *P19* and *P21* were shown in Supplementary Table 3. *GAPDH* was used as an endogenous reference for quantification.

**Fetal Alcian blue and Alizarin red staining**

Pregnant mice were sacrificed and fetuses (E16.5) and new born (P0) were collected. The gold standard Alizarin red/Alcian blue staining for observation of fetal bone development and mineralization was done according to a method published previously [8].

**Micro-computerized tomographic analysis**

Hind limbs and lungs from control and PM_2.5_-exposed offspring were scanned using a desktop scanner (1076 Skyscan Micro-CT; Skyscan, Kontich, Belgium) and analyzed with CTAn software (Skyscan).

**Blood test**

PB samples from control and PM_2.5_-exposed offspring were collected in Vacutainer (BD Biosciences) plastic tubes coated with K_2_EDTA and the numbers of WBCs, RBCs, and platelets measured with an automated complete cell counter (PE-6800VET; PROKAN).

**Immunohistochemistry analyses**

Paraffin-embedded 5 µm tissue sections of fetal and pup lungs, Ln, Sp, liver, and decalcified pup femur were subjected to IHC staining to detect the levels of COX-2 (BS1076; Bioworld Technology), TNF-α (BS1857; Bioworld Technology), 8-OHdG (sc-66036; Santa Cruz Biotechnology), CD4 (#25229; Cell Signaling Technology), CD8 (sc-7970; Santa Cruz Biotechnology), NLRP3 (ab214185; Abcam), IL-1β (sc-52012; Santa Cruz Biotechnology), MPO (ab9535; Abcam), RANKL (Bioworld Technology), and osteocalcin (OCN, BS7961, Bioworld Technology) using an IHC Accessory Kit (Vector Laboratories, Burlingame, CA, USA). Briefly, tissue sections were deparaffinized with xylene and rehydrated. Endogenous peroxidase was quenched by incubating sections in 0.3% H_2_O_2_ in methanol for 30 min at room temperature and thoroughly washed in buffer for 5 min followed by blocking the sections for 30 min with diluted normal blocking serum (which was prepared from the species in which the secondary antibody was made). Primary antibodies were diluted in buffer and sections were incubated overnight at 4 °C. Sections were again washed with buffer for 5 min and incubated for 30 min with diluted biotinylated secondary antibody solution. Following washing the slide with buffer, sections were incubated for 30 min with VACTASTAIN® ABC reagent followed by incubating the sections in peroxidase substrate solution until the desired stain intensity developed. Finally, the slides were counterstained with Meyer’s hematoxylin and observed using a light microscope (EL-Einsatz 451888; Carl Zeiss, Ostalbkreis, Germany).

**TRAP staining**

Mice femurs were fixed in a 4% paraformaldehyde solution for 24 h and decalcified in 10% EDTA solution at 4°C for 4 weeks. The decalcified specimens were dehydrated, embedded in paraffin, and sectioned at a thickness of 5.0 µm. A part of each tissue section was subjected to TRAP staining using a leukocyte acid phosphatase kit (Sigma-Aldrich). Tissue sections were counterstained with hematoxylin. Multinucleated TRAP-positive cells with a pink red color lining on the surface of trabecular bone were determined to be active osteoclasts.

**Osteoclast differentiation, in vitro TRAP staining**

For osteoclast differentiation, BM cells were obtained from control or PM_2.5_-exposed offspring, and seeded into 48-well culture plates at a density of 10,000 cells per well in the presence of 30 ng/mL murine M-CSF and 50 ng/mL murine RANKL (PeProtech, USA). The culture medium was replaced with a fresh batch of the same media on day 3. After 5 d of incubation, the cultures were fixed in 4% PBS-buffered paraformaldehyde and stained with a TRAP Staining Kit (Sigma-Aldrich) in accordance with the manufacturer’s instructions. TRAP-positive cells were counted by optical microscopy, and cells containing three or more nuclei were considered osteoclasts.

**Western blot analysis**

Briefly, whole protein lysates obtained from the BM of either the control or PM_2.5_-exposed offspring and protein extracts (10–20 µg per sample) were separated by sodium dodecyl sulfate-polyacrylamide gel electrophoresis on 8–12% gels and electroblotted onto polyvinylidene difluoride membranes. Blots were washed with a buffer containing 10 mM Tris-HCl (pH 7.6), 150 mM NaCl and 0.05% Tween-20 and then blocked in 5% skim milk for 1 h before incubation with each of primary antibodies. Membranes were washed and exposed to horseradish peroxidase-conjugated goat anti-rabbit IgG or goat anti-mouse IgG. Immunoreactive bands were visualized by enhanced peroxidase detection kit (ELPIS-Biotech, Taejeon, Korea) followed by exposure to X-ray film (Eastman Kodak, Rochester, NY, USA).

Western blot analyses were conducted using the following antibodies: Caspase-1 (#2225; Cell Signaling Technology), RUNX2 (BS2831; Bioworld Technology), osterix (ab94744; Abcam), osteopontin (ab8448; Abcam), RANKL (BS1867; Bioworld Technology), MMP-2 (BS1236; Bioworld Technology), MMP-9 (BS6893; Bioworld Technology), cathepsin-K (sc-48353; Santa Cruz Biotechnology), γ-H2AX (ab26350; Abcam), and p21 (sc-817; Santa Cruz Biotechnology). The β-actin antibody (sc-47778; Santa Cruz Biotechnology) was used as an internal control.

**ELISA**

The Multi-Analyte ELISArray Kits were used according to the manufacturer’s instructions (QIAGEN Sciences, Maryland, USA). Briefly, blood serum samples from control or PM_2.5_-exposed offspring were collected and diluted with sample dilution buffer. Experimental samples, along with the standard, were transferred to a 96-well plate (pre-coated Capture Antibody Microplate) and incubated for 2 h at room temperature. Following washing with 1x wash buffer, avidin-HRP solution was added to all wells and incubated for a further 30 min at room temperature. Development solution was added to each well following incubation of the plate for 15 min at room temperature in the dark. Finally, a stop solution was added and the color change (from blue to yellow) was recorded by measuring the absorbance at 450 nm using a microplate reader (Packard Instrument Co., Downers Grove, IL, USA) within 30 min of stopping the reaction.

**In vitro proliferation activity**

The cell proliferation activity of BM cells obtained from either control or PM_2.5_-exposed offspring was determined with the WST-8 reagent using the Cell Counting Kit-8 (Dojindo Molecular Tech. Inc., Kumamoto, Japan). In brief, BM cells (5000/well) were seeded in a 96-well plate and incubated in the plate for 1–4 d. The cells were treated with WST-8 reagent each day during the incubation period, followed by incubation for an additional 2 h. Finally, WST-8-specific absorbance was measured at 450 nm using a microplate reader (Packard Instrument Co., Downers Grove, IL, USA).

**Bone mineralization assay**

BM cells obtained from control and PM_2.5_-exposed offspring were incubated in presence or absence of an osteogenic medium (α-minimum essential medium containing 5% FBS, 50 μM ascorbic acid, 100 nM dexamethasone and 10 mM β-glycerophosphate) for 21 d. To determine the degree of mineralization, Alizarin Red staining was performed. In brief, cells were fixed for 30 min in 4% paraformaldehyde and washed three times with ice-cold PBS. The cells were stained for 5 min with Alizarin Red, followed by observation under a light microscope. To quantify the amount of red dye, the stain was eluted with 10% acetylpyridinum chloride by shaking for 20 min and the absorbance measured at 570 nm.

**Statistical analyses**

All data are expressed as the mean ± standard deviation (SD). The Student’s *t-*test was used to determine significant differences between two sets of data, and one-way analysis of variance was used for multiple comparisons using SPSS version 16.0 software (Chicago, IL, USA). P < 0.05 was considered statistically significant.

**References**

1. Braban CF, Abbatt JPD. A study of the phase transition behavior of internally mixed ammonium sulfate-malonic acid aerosols. Atmos Chem Phys. 2004; 4: 1451–1459.

2. Decesari S, Fuzzi S, Facchini MC, Mircea M, Emblico L, Cavalli F, et al. Characterization of the organic composition of aerosols from rondonia, Brazil, during the LBA-SMOCC 2002 experiment and its representation through model compounds. Atmos Chem Phys. 2006; 6: 375–402.

3. Ling TY, Chan CK. Partial crystallization and deliquescence of particles containing ammonium sulfate and dicarboxylic acids. J Geophys Res Atmos. 2008; 113: D14205.

4. Gilardoni S, Liu S, Takahama S, Russel LM, Allan JD, Steinbrecher R, et al. Characterization of organic ambient aerosol during MIRAGE 2006 on three platforms. Atmos Chem Phys. 2009; 9: 5417–5432.

5. Hawkins LN, Russel LM, Covert DS, Quinn PK, Bates TS. Carboxylic acids, sulfates, and organosulfates in processed continental organic aerosol over the southeast Pacific Ocean during VOCALS-REx 2008. J Geophys Res Atmos. 2010; 115: D13201.

6. Pauluhn J, Mohr U. Inhalation studies in laboratory animals-current concepts and alternatives. Toxicol Pathol. 2000; 28: 734–753.

7. Ye J, Salehi S, North ML, Portelli AM, Chow CW, Chan AW. Development of a novel simulation reactor for chronic exposure to atmospheric particulate matter. Sci Rep. 2017; 7: 42317.

8. Rigueur D, Lyons KM. Whole-mount skeletal staining. Methods Mol Biol. 2014; 1130: 113-121.

**Supplementary Figure’s Legends**

**Supplementary Fig. 1** Maternal exposure to fine PM_2.5_ during pregnancy impairs the lungs of the fetus and offspring by inducing oxidative stress and inflammation. **a** Schematic representation of the ASC system used to expose the mice to PM_2.5_. **b,c** Characterization of PM in an exposure chamber without mice using the OPC: time evolution of PM concentrations (**b**) and normalized number concentration of PM versus PM optical diameters (**c**). Representative data are shown from three independent experiments. **d,e** The expression levels of COX-2, TNF-α, and 8-OHdG were immunohistochemically measured in the lung of E16.5 after maternal exposure to PM_2.5_ for 2 h on five consecutive days at E12.5 (**d**, n = 5) and in that of offspring (2 months old) born in their dam that had already been exposed to PM2.5 for the designated period (**e**, n = 5); a representative result is shown. Scale bars are 100 µm. DAB-positive cell intensity was measured by ImageJ-win64. **f** Expression of CD4 and CD8 in lungs tissue of control and PM_2.5_-exposed mice was analyzed by IHC. A representative result is shown (n = 8). Scale bars are 100 µm. **g** Collagen synthesis due to the inflammation in mice lung’s was examined by Masson’s Trichrome staining. A representative result is shown (n =7). Scale bars are 50 µm. **h** Expression levels of NLRP3 and IL-β in lung tissues of control and PM_2.5_-exposed offspring were analyzed by IHC. **i** Expression level of Caspase-1 in lung tissues of control and PM_2.5_-exposed offspring was analyzed by western blotting. A representative result is shown (n = 4) All data are presented as mean ± SD. ***p < 0.001 vs. control, as determined by Student’s *t*-tests.

**Supplementary Fig. 2** Effect of maternal exposure to fine PM_2.5_ during pregnancy in various tissues of the fetus and offspring. **a** maternal exposure to fine PM_2.5_ during pregnancy affects various tissues in the fetus and offspring. The expression levels of 8-OHdG, COX-2, and TNF-α were immunohistochemically measured in the liver and brain of E16.5 after maternal exposure to PM_2.5_ for 2 h for five consecutive days at E12.5 (n = 6) and the liver, brain, kidney, thymus, and spleen of offspring (2 months old) born to dams that had already been exposed to PM2.5 for the designated period (n = 5); a representative result is shown, scale bars are 100 µm. **b,c** Whole-mount staining with Alizarin Red and Alcian Blue of E16.5 embryo and P0 bones. The lengths of the Alizarin Red stained portions with calcified tissue were indicated by red bracket (n = 3); a representative result is shown.

**Supplementary Fig. 3** Maternal exposure to fine PM_2.5_ during pregnancy does not directly affect HSCs in the FL of E16.5d and the BM of offspring of 2 months. **a** Number of FL HSCs, phenotypically defined by Lineage^-^Mac-1^+^Sca-1^+^CD150^+^CD48^-^ cells, was measured in E16.5 that had been maternally exposed to PM_2.5_ by multicolor flow cytometry (n = 8). **b** Clonogenic formations of CFU-GM, BFU-E, and CFU-GEMM after 12 days of incubation in a methylcellulose-based medium with FL cells (2 × 10^4^ per dish); representative data are shown for three independent experiments. **c** BM cellularity was measured from control and PM_2.5_-exposed offspring of 2 months (n = 5). **d** Number of BM HSCs, phenotypically identified by Lineage^-^Sca-l^+^c-Kit^+^CD150^+^CD48^-^ cells, measured in offspring of 2 months, born in their dam exposed to PM2.5 (n = 10). **e** The numbers of colonies formed by the incubation of BM cells (2 × 10^4^ per dish) of the PM_2.5_-exposed offspring were countered; representative data are shown from three independent experiments. **f** Numbers of WBCs, RBCs and platelets in the PB of the offspring were measured with an automated complete cell counter (n = 7). All data are presented as the means ± SD.

**Supplementary Fig. 4** Functional and differentiation analyses of HSCs in PM_2.5_-exposed offspring of 6 months. **a** BM cells (1 × 10^6^) from control or PM_2.5_-exposed offspring at 6 months were non-competitively transplanted into lethally irradiated recipient animals (n = 10). The ability of the donor cells to support survival of the recipient animal for 4–5 weeks was attributed to short-term repopulating cells (radioprotection potential). Survival beyond 16 weeks demands long-term repopulating cells. **b** Survival rate of control and PM_2.5_-exposed offspring were measured after exposure to a sublethal dose (5 Gy) of total body irradiation (n = 10). **c** BM cellularity was measured from control and PM_2.5_-exposed offspring (n = 5). **d,e** Levels of ERK (**d**) and JNK (**e**) phosphorylation were analyzed in the BM HSCs of the offspring after the fixation and permeabilzation procedure (n = 7). **f** The number of BM HSCs was analyzed in the offspring (n = 11). **g** Frequency of hematopoietic progenitor cells in the BM of the offspring was measured using FcR and CD34 antibodies in a basis of Lin^-^Sca-1^-^c-Kit^+^ markers, and IL-7R antibody (n = 8, GMP, granulocyte–monocyte progenitors; CMP, common myeloid progenitors; MEP, megakaryocyte–erythroid progenitors; CLP, common lymphoid progenitors). **h** Proportion of circulating monocytes and B lymphocytes in the PB of the offspring were measured using staining the cells with CD11b and B220 antibodies, respectively (n = 8). **i** mRNA levels of *p15, p16, p19,* and *p21*, whose primer sequences were mentioned in Supplementary Table 3, were measured in CD150^+^CD48^-^LSK cells sorted from the BM of control and PM2.5-exposed offspring; a representative result is shown from three independent experiments. **j** Numbers of WBCs, RBCs and platelets in the PB of the offspring were measured with an automated complete cell counter (n = 13). All data are presented as the means ± SD. **p < 0.01 and ***p < 0.001 vs. control, as determined by Student’s *t*-tests.

**Supplementary Fig. 5** Maternal exposure to fine PM_2.5_ during pregnancy leads to the preferential impairment of the BM microenvironment by inducing the senescence of bone cells and MSCs in offspring of 2 months. **a** For micro-computerized tomographic analysis, hind limbs from control and PM_2.5_-exposed male offspring of 2 months were scanned using a desktop scanner and bone parameters was analyzed with CTAn software. Representative data are shown (n = 5). BV/TV,bone volume/tissue volume; Tb.N, trabecular number; Tb.Th, trabecular thickness; Tb.Sp, trabecular separation; Conn.Dn, Connectivity density; BMD, bone mineral density. **b** Expression level of osteocalcin (OCN) was immunohistochemically measured in the femur of control and PM_2.5_-exposed offspring; a representative result is shown (n = 4). Scale bars are 50 µm. **c** Proliferative capacity of BM cells was measured at the indicated times using a CCK-8 kit; representative data are shown from four independent experiments. **d** ROS levels were measured in BM cells (passage 3) cultured from control or PM_2.5_-exposed offspring of 2 months; representative data are is shown from four independent experiments. **e** BM cells at passage 3 were subjected to SA-β-gal staining (left) and the number of SA-β-gal-positive cells was counted. At least 50 cells from 4 random fields were counted. It should be noted that BM cells of PM_2.5_-exposed offspring showed a senescence-like morphology, (e.g., a large and flattened); a representative result is shown from three independent experiments. Red arrows indicate SA-β-gal-stained cells. Scale bars are 20 µm. **f** The expression levels of γ-H2AX and p-21 proteins were assessed in BM cells flushed from the offspring using western blotting, a representative result is shown from three independent experiments. The band intensities were quantified with ImageJ densitometry software (National Institutes of Health, Bethesda, MD, USA). **g** BM cells were incubated in the presence or absence of osteogenic differentiation media. After 5 days of incubation the expression levels of osteogenic molecules were analyzed by Western blotting; a representative result is shown from three independent experiments. The band intensities were quantified with ImageJ densitometry software (National Institutes of Health, Bethesda, MD, USA). **h** After 21 days of incubation BM cells were stained with Alizarin red and the optical density specific to the dye was measured at 570 nm; a representative result is shown from three independent experiments. **i** Representative schematic diagram of the flow cytometry gating strategy used to phenotypically define BM MSC populations using Lin^-^Sca-1^+^CD29^+^CD105^+^ markers (left). The number of BM MSCs were measured in control and PM_2.5_-exposed offspring at 2 months of age (n = 10). **j–k** Analyses of mitochondrial ROS levels (**j**, n = 10) and SA-β-gal activity (**k**, n = 10), in BM MSCs of the offspring. **l** The colony-forming activities of BM cells derived from the offspring were compared by counting the number of colonies formed 14 days after incubation with BM nucleated cells (1 × 10^6^); representative data are shown from three independent experiments. All data are presented as the means ± SD. *p < 0.05, **p < 0.01 and ***p < 0.001 vs. control, as determined by Student’s *t*-tests and ^#^p < 0.05, ^##^p < 0.01 and ^###^p < 0.001 vs. 1 days as determined by one-way analysis of variance using SPSS software (ver.12.0) for multiple comparisons.

**Supplementary Fig. 6** Maternal exposure to fine PM_2.5_ during pregnancy provokes osteoclastogenesis in the BM of offspring of 2 months. **a** Osteoclasts in the BM of control and PM_2.5_-exposed offspring of 2 months were visualized by staining the cells with TRAP and counted in three sections per bone. Arrows indicate TRAP-positive osteoclasts. Scale bars are 50 µm. A representative result is shown (n = 5). **b** The intensity of RANKL protein was immunohistochemically analyzed in the BM of the offspring; a representative result is shown (n = 5). Scale bars are 100 µm. **c** The expression level of RANKL protein was assessed in BM cells flushed from the offspring using Western blotting; a representative result is shown from three independent experiments. **d** The levels of RANKL and OPG in cell-free BM supernatant of the offspring were measured using a sandwich ELISA kit; representative data are shown from three independent experiments. **e** The levels of pro-inflammatory cytokines in blood serum of the offspring were measured using a Multi-Analyte ELISArray Kit; representative data are shown for three independent experiments. **f** Proportion of monocyte/macrophage cells, osteoclast precursor cells, was analyzed in the BM of the offspring by incubating the cells with a CD11b antibody (n = 6). **g** BM cells from the offspring were treated with an osteoclast differentiation medium as mentioned in the Materials and Methods section of this supplementary file and were stained using a TRAP assay. Multinucleated cells (i.e., those with more than three nuclei) were imaged and counted; representative data shown from three independent experiments. Scale bars are 500 µm. **h** The expression levels of proteolytic enzymes were analyzed by Western blotting in BM cells flushed from the offspring; a representative result is shown from three independent experiments. The band intensities were quantified with ImageJ densitometry software (National Institutes of Health, Bethesda, MD, USA). All data are presented as the means ± SD. *p < 0.05, **p < 0.01 and ***p < 0.001 vs. control, as determined by Student’s *t*-tests.

**Supplementary Fig. 7** The BM of PM_2.5_-exposed offspring is not conducive to the engraftment and functional integrity of HSCs. **a** Schematic diagram of the transplantation protocol. Normal BM cells (2 × 10^6^ per recipient, CD45.1/2) were transplanted into 8-week-old control or PM2.5-exposed offspring (CD45.2) that had been irradiated with 3 Gy. **b** For donor cell-derived reconstituting ability, PB were collected in the conditioned recipients at 4, 8 and 16 weeks post-transplant and analyzed using the mixture of CD45.1 and CD45.2 antibodies (n = 6). **c–f** At 16 weeks post-transplant, the percentage (**c**, n = 5), mitochondrial ROS level (**d**, n = 5), p38 phosphorylation (**e**, n = 5) and SA-β-gal activity (**f**, n = 5) of HSCs derived from donor cells (CD45.1/2) were analyzed in the BM of the conditioned recipients. **g** Clonogenic formations of CFU-GM, BFU-E, and CFU-GEMM after 12 days of incubation in a methylcellulose-based medium with BM cells (2 × 10^4^ per dish) engrafted in the conditioned offspring recipients at 16 weeks post-transplant; representative data are shown from three independent experiments. All data are presented as the means ± SD. *p < 0.05, **p < 0.01 and ***p < 0.001 vs. control, as determined by Student’s *t*-tests.

**Supplementary Fig. 8** Adolescent PM_2.5_-exposed mice are resistant to BM microenvironment-mediated HSC senescence. **a** The expression levels of COX-2, TNF-α, and 8-OHdG in the lung of control and adolescent PM_2.5_-exposed mice at 6 months of age; a representative result is shown (n = 5). Scale bars are 50 µm. **b** Micro-computerized tomographic analyses were performed on hind limbs taken from the mice. Representative data are shown (n = 4). BV/TV,bone volume/tissue volume; Tb.N, trabecular number; Tb.Th, trabecular thickness; Tb.Sp, trabecular separation; Conn.Dn, Connectivity density; BMD, bone mineral density. **c** TRAP-positive osteoclasts in the trabecular region of the mice were counted in three sections per bone. Scale bars are 200 µm. A representative result is shown (n = 5). **d** The number, mitochondrial ROS level and SA-β-gal activity of MSCs were assessed in the BM of the mice (n = 5). **e** The number, mitochondrial ROS level, p38 phosphorylation and SA-β-gal activity of HSCs were assessed in the BM of the mice (n = 5). All data are presented as the means ± SD.

**Supplementary Fig. 9** SA-β-gal activity in BM MSCs and HSCs of PM_2.5_-exposed offspring at 1 year of age more increases. The levels of SA-β-gal activity were measured in BM MSCs and HSCs of PM_2.5_-exposed offspring at 1 year of age (n = 5). All data are presented as the means ± SD. ***p < 0.001 vs. control, as determined by Student’s t-tests.

**Supplementary Fig. 10** Maternal PM_2.5_-exposed offspring exhibit myeloproliferative disease-related phenotypes. **a** BM and Sp cellularity were measured from old control and PM_2.5_-exposed offspring (n = 4). **b** Percentage of immature and mature myeloid cells in PB, BM, and Sp was measured using c-Kit, Gr-1, and Ly6G antibodies (n = 4). **c** Percentage of T cells such as CD4^+^ and CD8^+^ cells in Sp of the old offspring (n = 5). All data are presented as the means ± SD. ***p < 0.001 vs. control, as determined by Student’s *t*-tests.

**Supplementary Fig. 11** Oral supplementation with NAC is effective in preventing HSC senescence by improving the BM microenvironment of offspring that have been impaired by maternal exposure to fine PM_2.5_. **a** The expression levels of COX-2, TNF-α, 8-OHdG and NLRP3 were measured immunohistochemically in the lungs of offspring of 6 months maternally exposed to fine PM_2.5_ with or without NAC treatment; a representative result is shown (n = 5). Scale bars are 100 µm. **b** Micro-computerized tomographic analyses were performed on hind limbs from the offspring; A representative result is shown (n = 5). BV/TV, bone volume/tissue volume; Tb.N, trabecular number; Tb.Th, trabecular thickness; Tb.Sp, trabecular separation; Conn.Dn, Connectivity density; BMD, bone mineral density. **c** The protein levels of RANKL and OPG in cell-free BM supernatants of the offspring were measured using a sandwich ELISA kit; representative data are shown from three independent experiments. **d** The levels of pro-inflammatory cytokines in blood serum of the offspring were measured; representative data are shown for three independent experiments. **e** Osteoclasts in the BM of the offspring were visualized by staining the cells with TRAP and counted in three sections per bone. Arrows indicate TRAP-positive osteoclasts. Scale bars are 50 µm. A representative result is shown (n = 5). **f** The number, mitochondrial ROS level and SA-β-gal activity of MSCs were assessed in the BM of the offspring (n = 5). **g** The number, mitochondrial ROS level, p38 phosphorylation and SA-β-gal activity of HSCs were assessed in the BM of the offspring (n = 5). **h** The numbers of colonies, such as CFU-GM, BFU-E, and CFU-GEMM, formed by the incubation of BM (2 × 10^4^ per dish) of the PM_2.5_-exposed offspring with or without NAC were countered; representative data are shown from three independent experiments. All data are presented as means ± SD. *p < 0.05, **p < 0.01 and ***p < 0.001 vs. control, as determined by Student’s *t*-test and one-way analysis of variance using SPSS software (ver.12.0) for multiple comparisons.

**Supplementary Fig. 1**


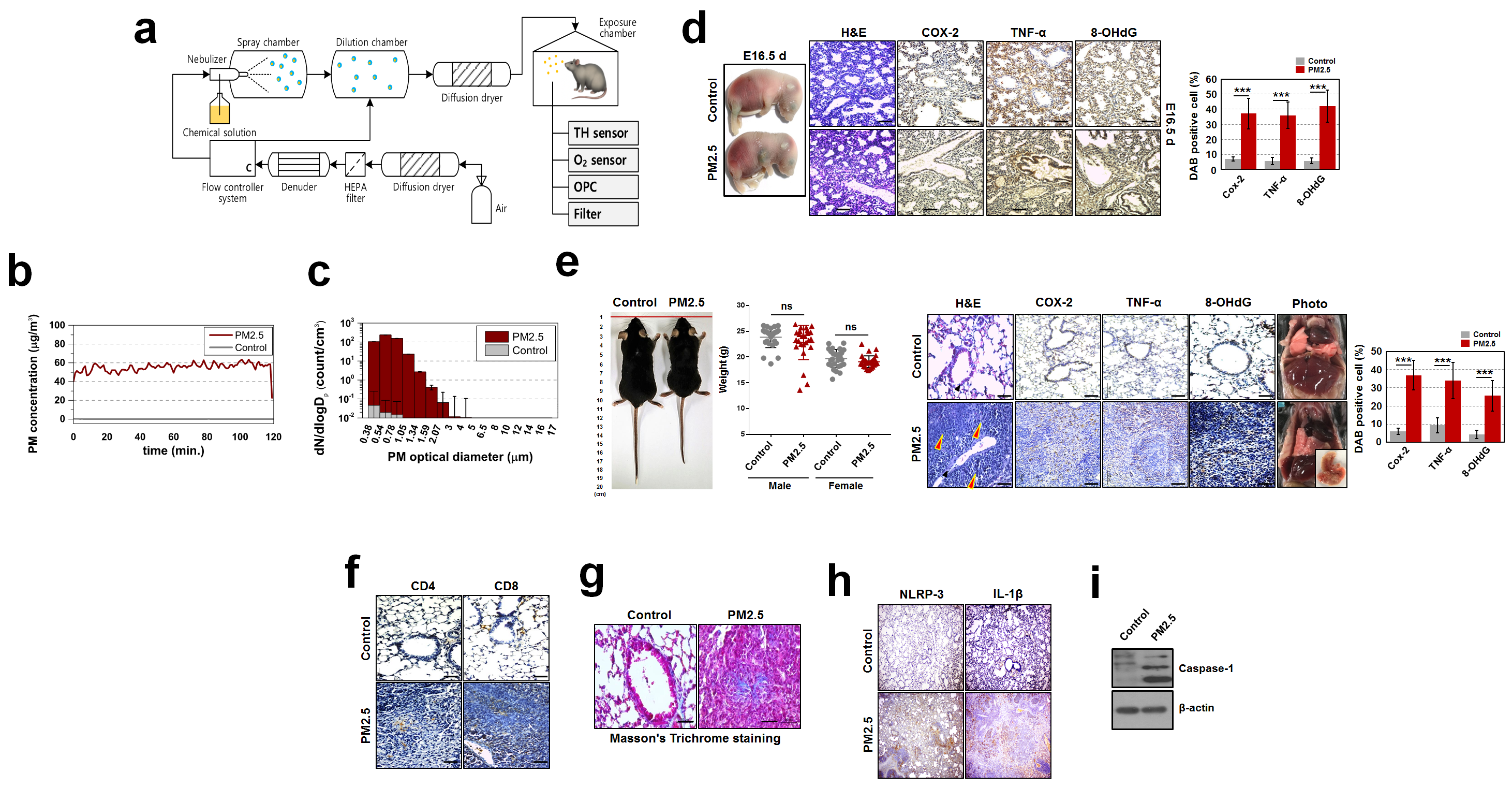


**Supplementary Fig. 2**


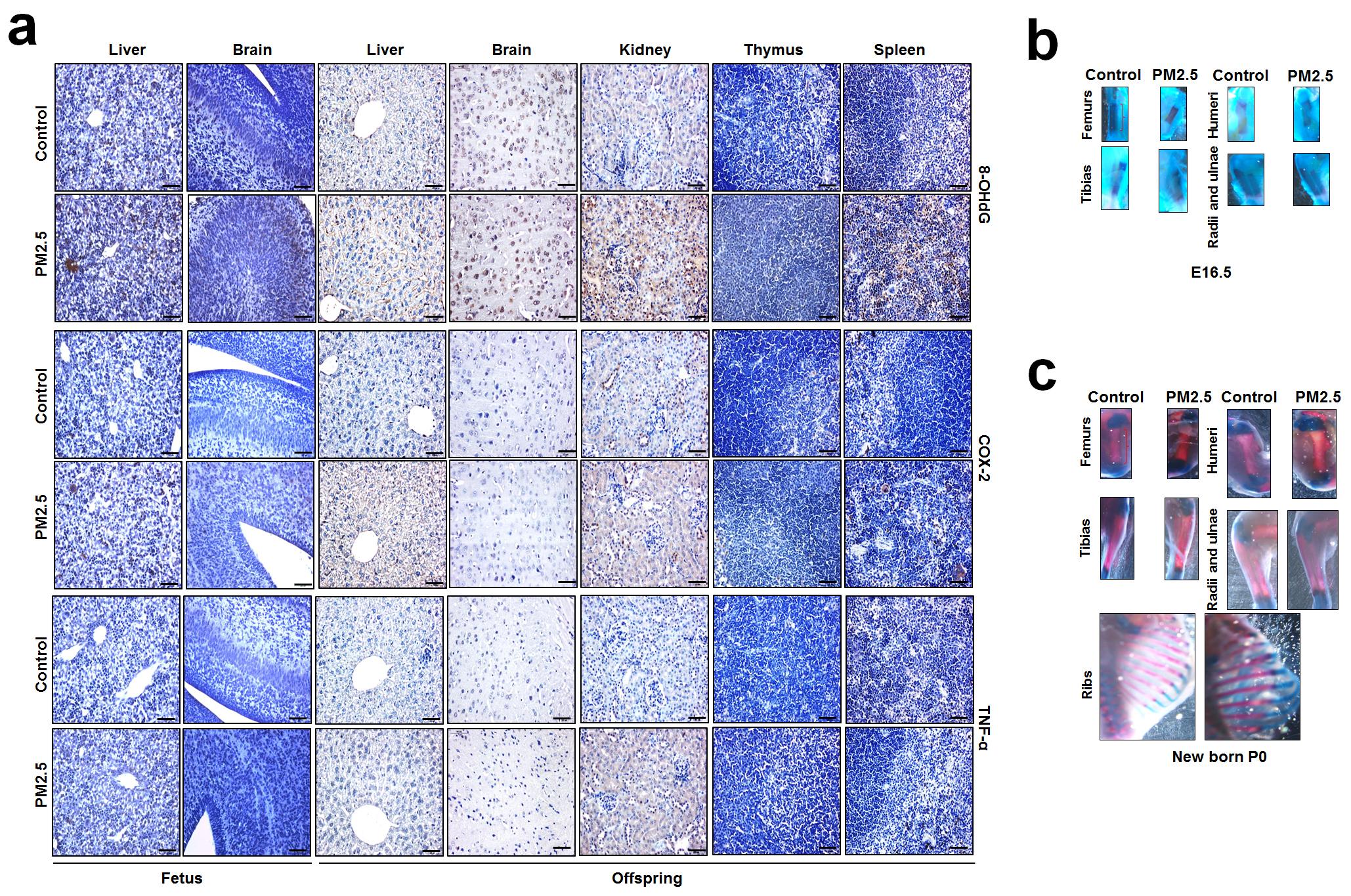


**Supplementary Fig. 3**


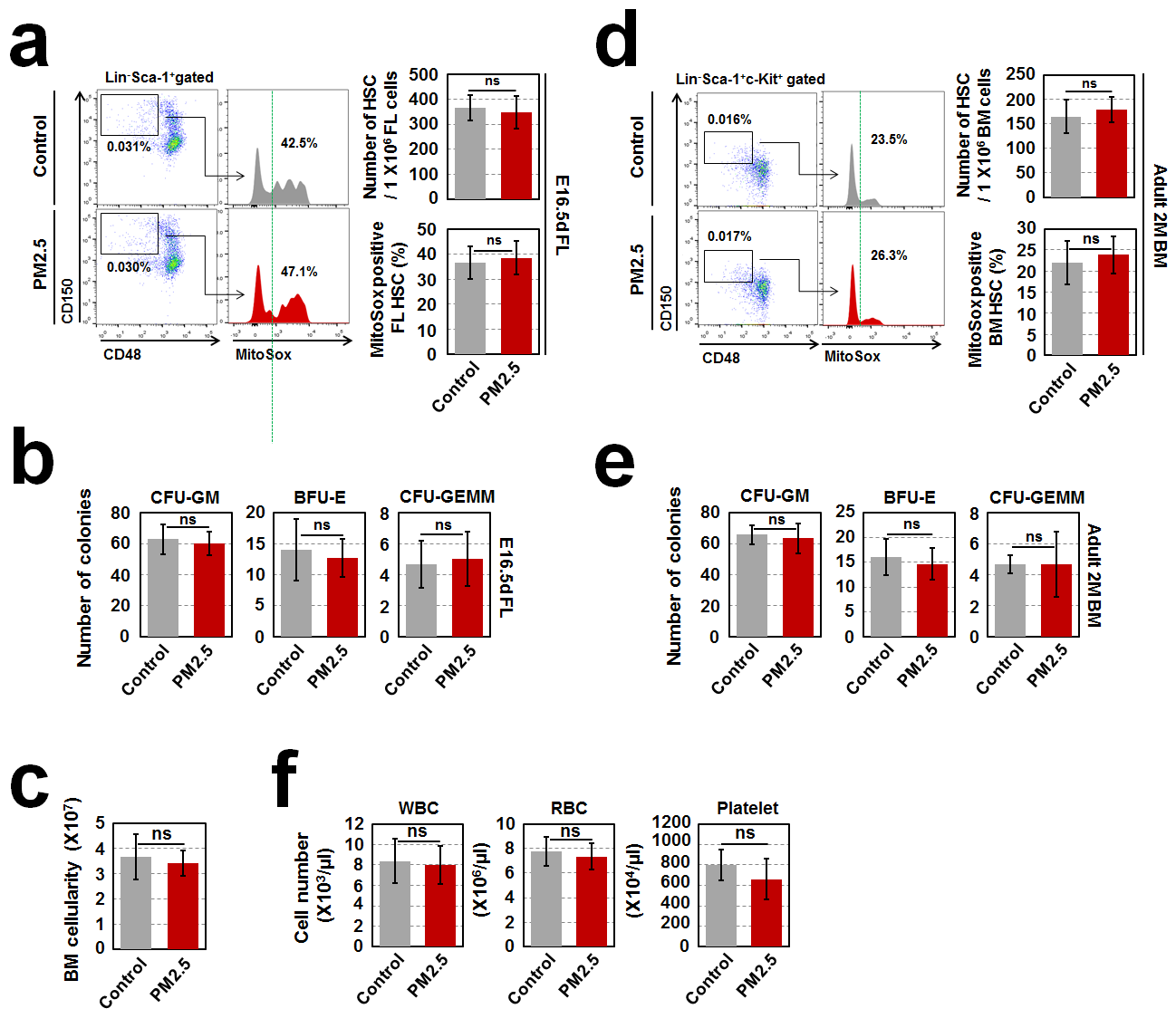


**Supplementary Fig. 4**

**
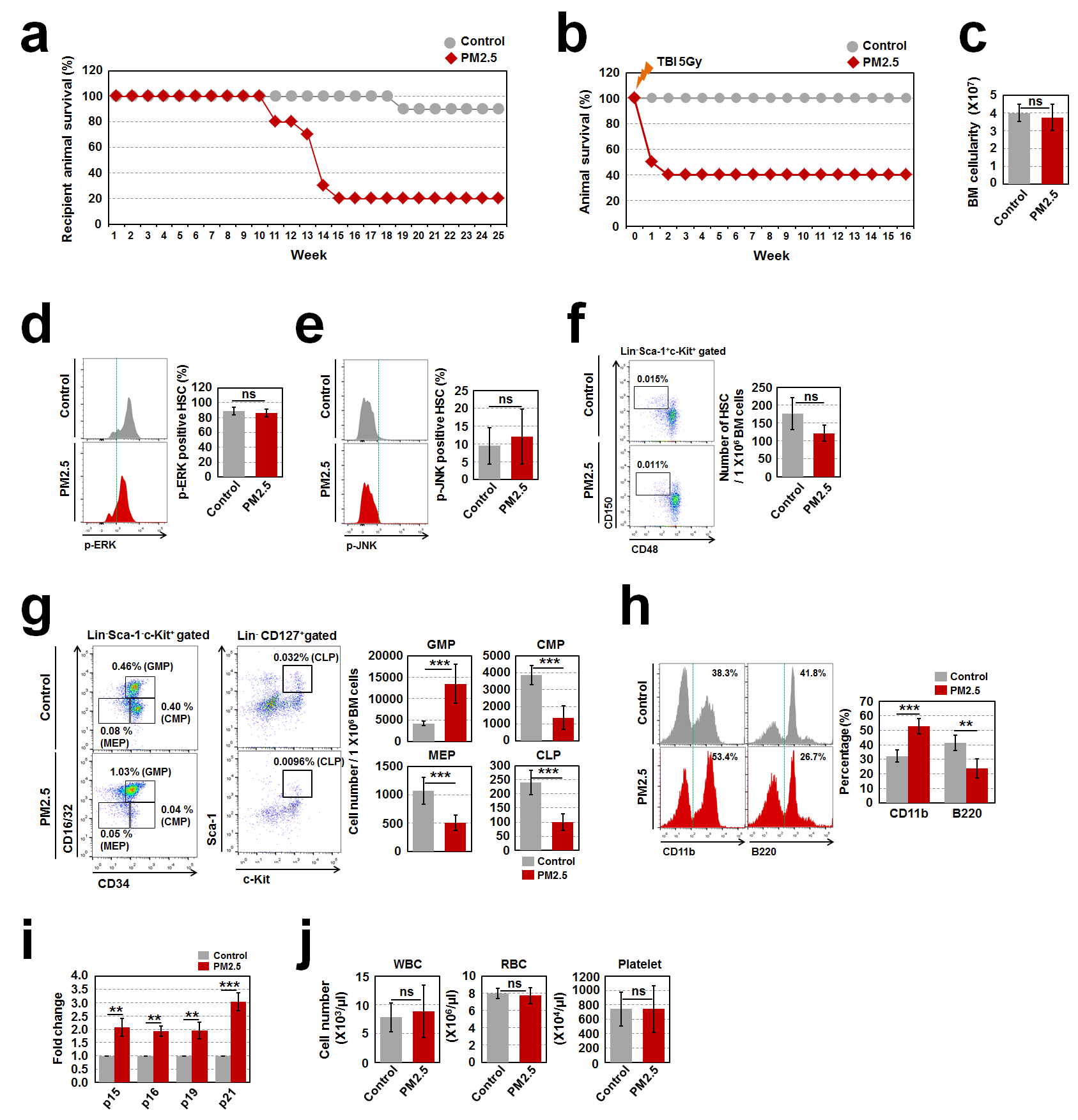
**

**Supplementary Fig. 5**


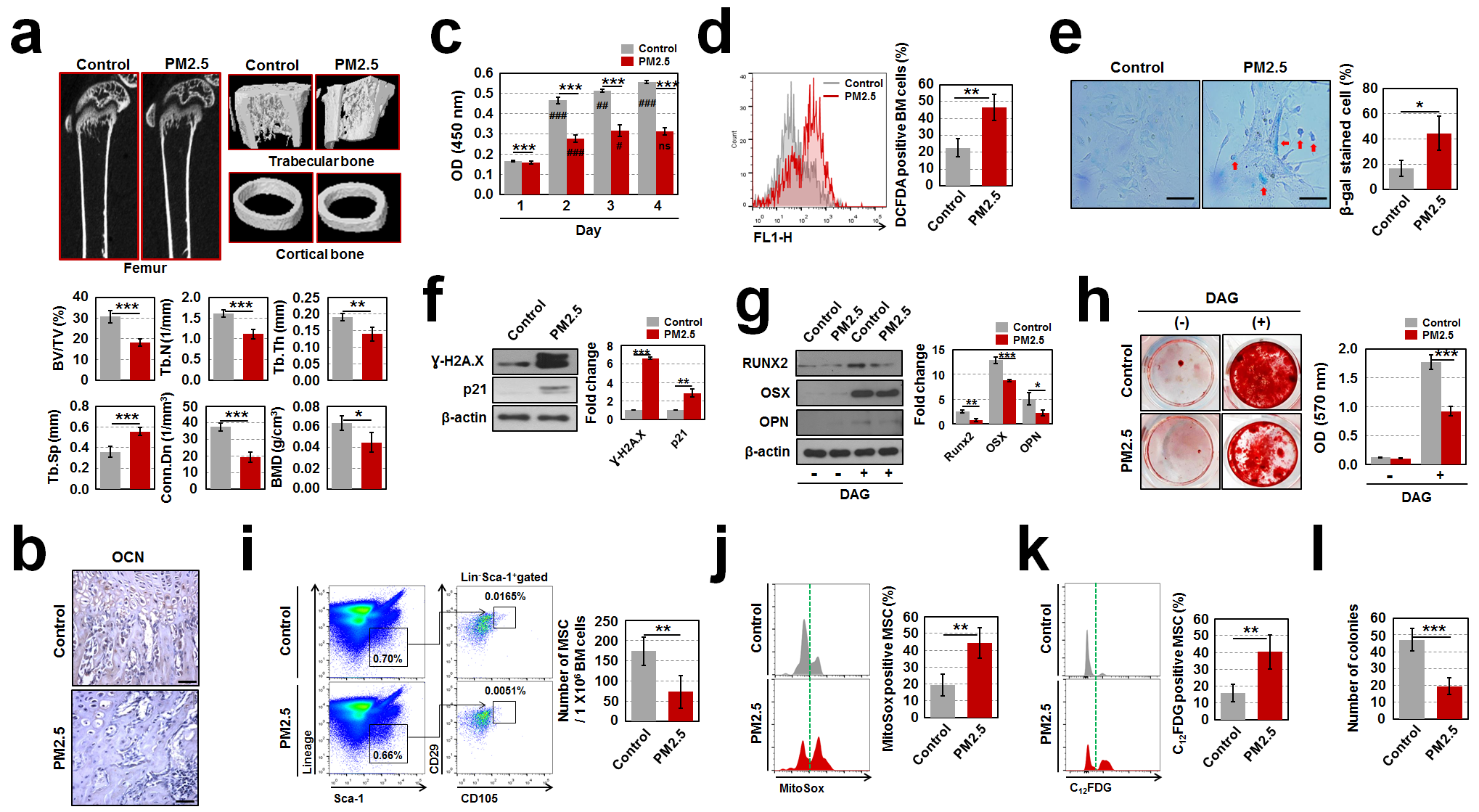


**Supplementary Fig. 6**


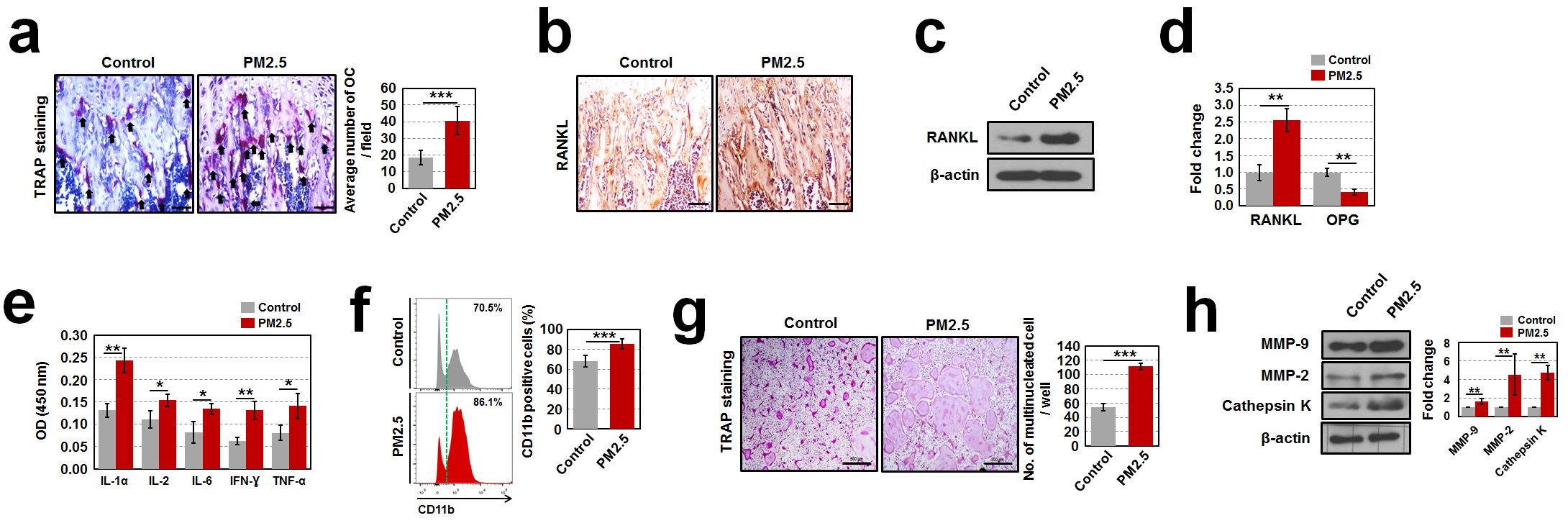


**Supplementary Fig. 7**


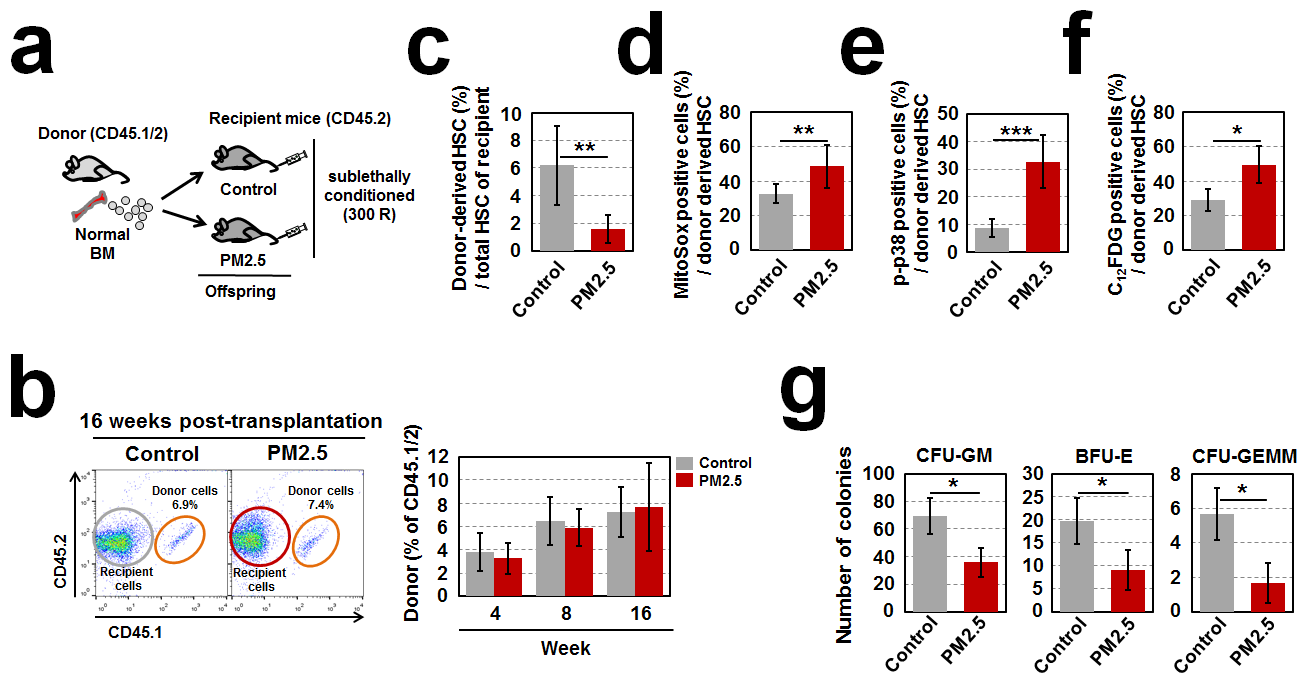


**Supplementary Fig. 8**


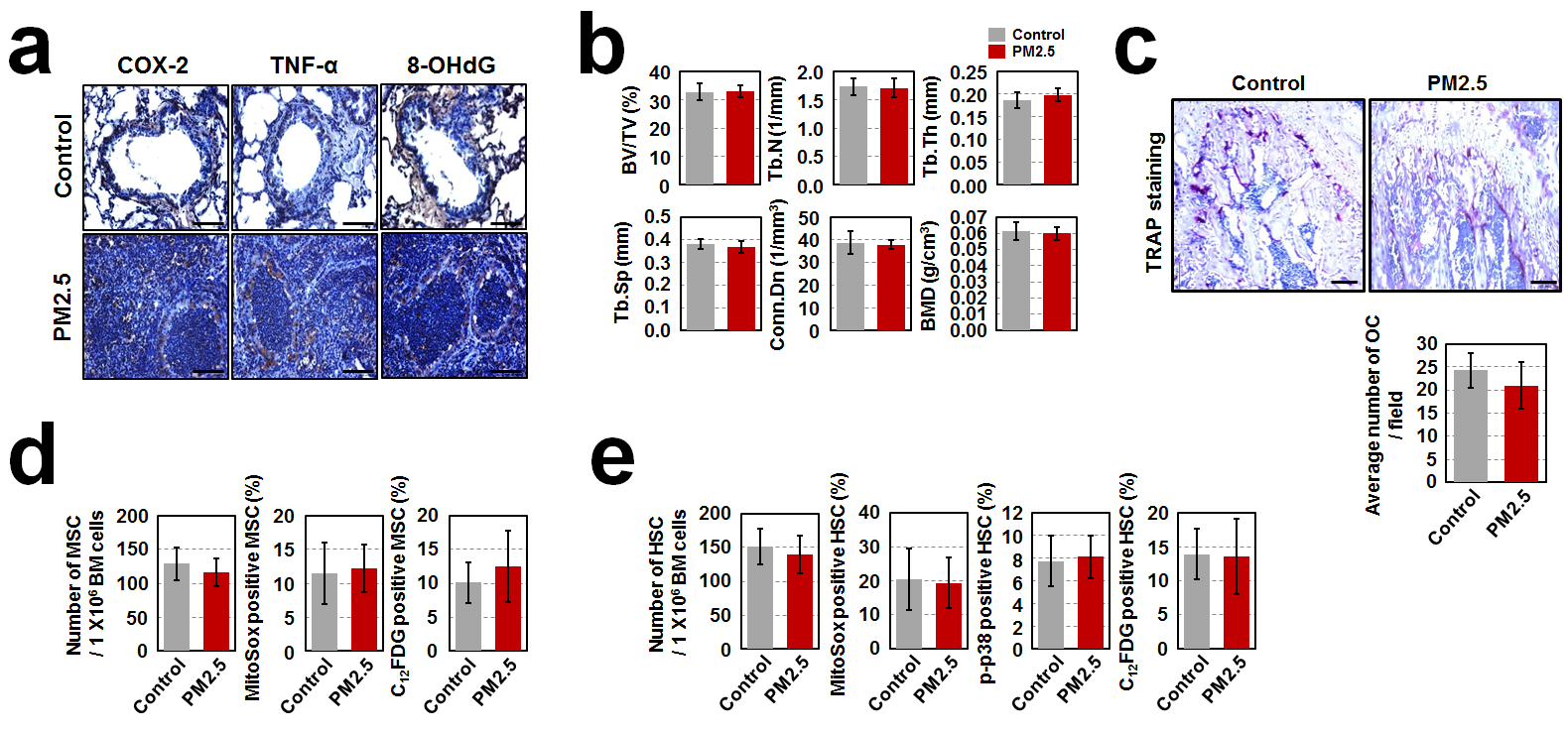


**Supplementary Fig. 9**

**
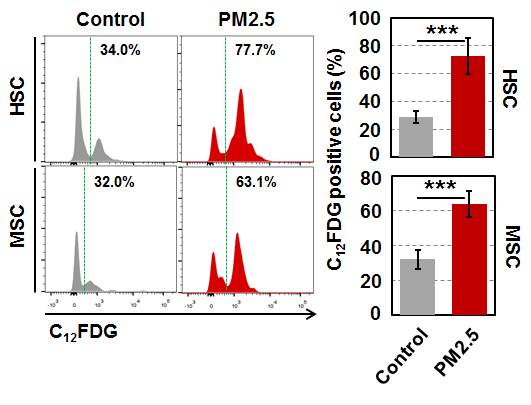
**

**Supplementary Fig. 10**

**
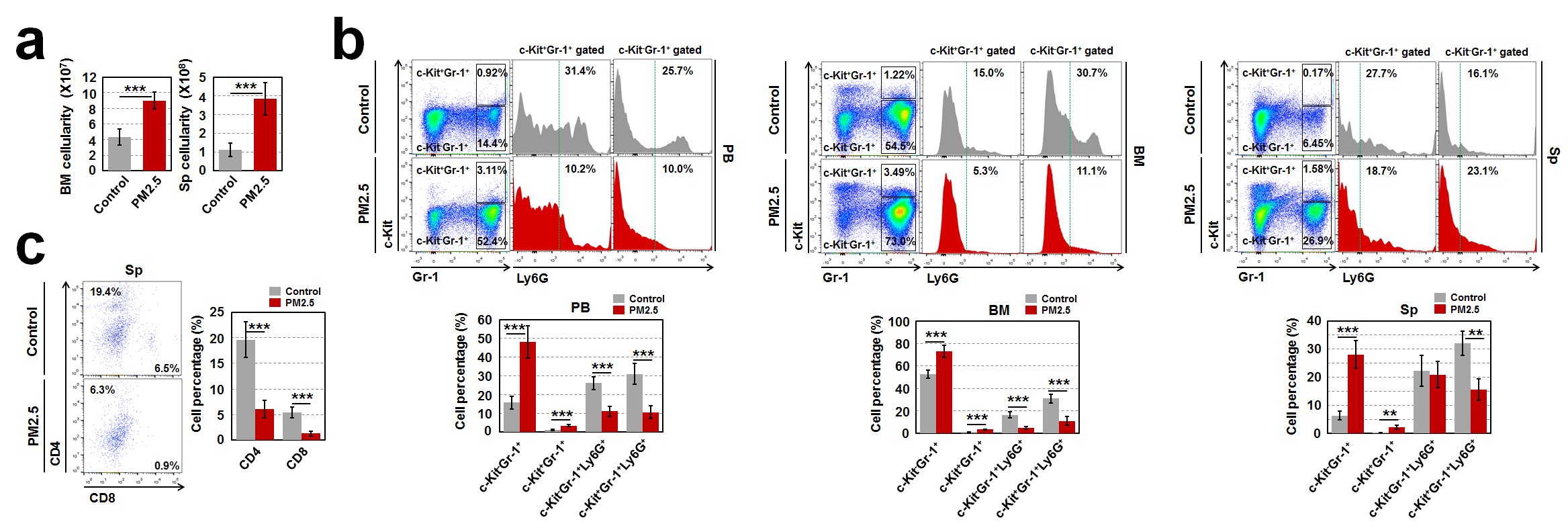
**

**Supplementary Fig. 11**


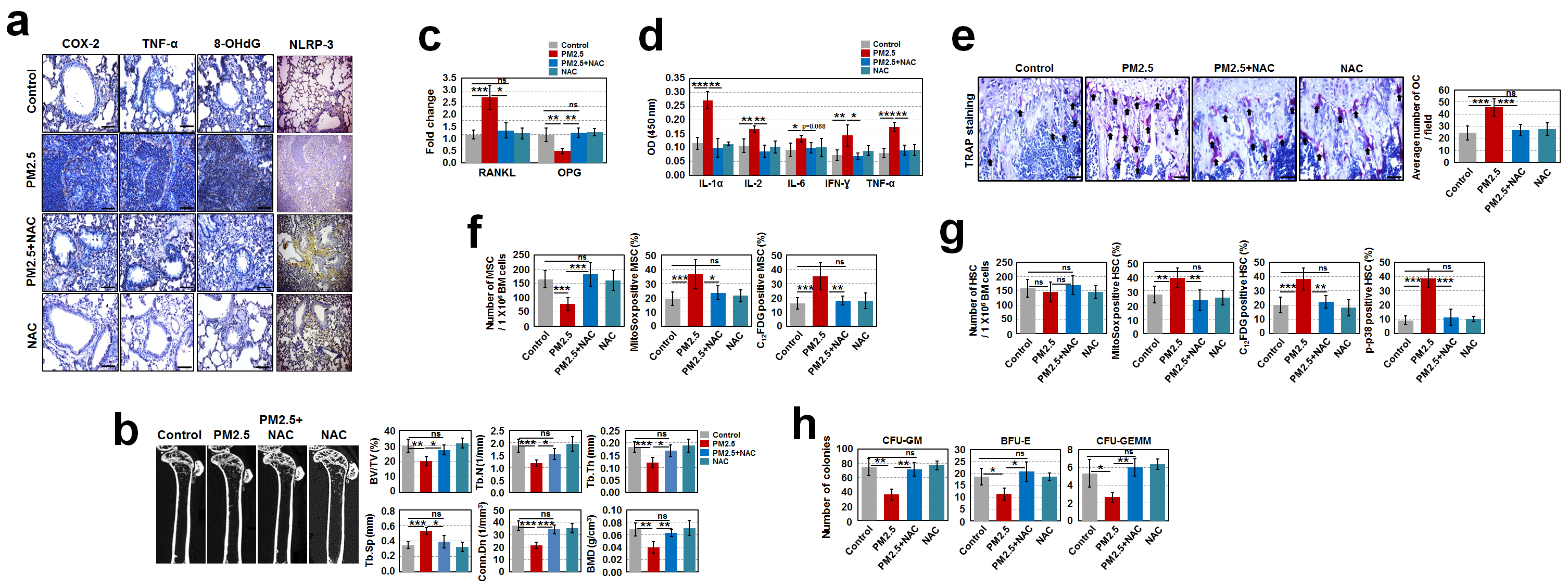


Supplementary Table 1. List of the chemical compositions, formula, and dry mass fractions of organic and inorganic species used in this study.

| **Functional Group** | **Components** | **Formula** | **Density (g/cm^3^) at 295 K^*^** | **Dry mass fraction (%)** |
| --- | --- | --- | --- | --- |
| Monocarboxylic acid | Acetate | C_2_H_3_O_2_ | 1.05 | 6.25 |
| Dicarboxylic acid | Oxalic acid | C_2_H_2_O_4_ | 1.90 | 6.25 |
|  | Malonic acid | C_3_H_4_O_4_ | 1.62 | 6.25 |
|  | Glutaric acid | C_5_H_8_O_4_ | 1.35 | 6.25 |
| Polyols | Glycerol | C_3_H_8_O_3_ | 1.26 | 6.25 |
| Sugars | Sucrose | C_12_H_22_O_11_ | 1.59 | 6.25 |
| Aromatics | 2,5-Dihydroxybenzoic acid | C_7_H_6_O_3_ | 1.55 | 6.25 |
| Amino acid | Glycine | C_2_H_5_O_2_N | 1.61 | 6.25 |
| Inorganic salts | Ammonium sulfate | (NH_4_)_2_SO_4_ | 1.77 | 25 |
|  | Ammonium nitrate | NH_4_NO_3_ | 1.72 | 25 |

^*^ Values of measured densities are from www.chemicalbook.com

Supplementary Table 2. Mean PM mass concentration in the exposure chamber

| Method | Filter (μg m^-3^) | OPC (μg m^-3^) |
| --- | --- | --- |
| Batch1 | 49.2 ± 5.2 | 47.0 ± 5.5 |
| Batch2 | 44.5 ± 10.4 | 53.0 ± 4.7 |
| Batch3 | 65.1 ± 3.0 | 66.5 ± 6.7 |

Supplementary Table 3. Primer sequences used in this study

| Gene | Forward sequence | Reverse sequence |
| --- | --- | --- |
| *p16* | GTCGCAGGTTCTTGGTCACT | TCTGCACCGTAGTTGAGCAG |
| *p21* | TGTCCGTCAGAACCCATC | AAAGTCGAAGTTCCATCGCC |
| *p15* | CCCTGCCACCCTTACCAGA | CAGATACCTCGCAATGTCACG |
| *p19* | GCCGGCAAATGATCATAGAG | CAGCAAGAGCTGGATCAGAA |
| *GAPDH* | GACGGCCGCATCTTCTTGT | CACACCGACCTTCACCATTTT |
